# 2146 Training cycle in clinical and translational research (CTR) for undergraduate health sciences programs (HSUP) at University of Puerto Rico-Medical Sciences Campus (UPR-MSC) and Universidad Central del Caribe (UCC): Pathway for students and faculty

**DOI:** 10.1017/cts.2018.232

**Published:** 2018-11-21

**Authors:** Margarita Irizarry-Ramírez, Rubén G. García, Edgardo L. R. Santiago, Juan C. S. Santiago, Carlamarie Noboa, José Moscoso-Álvarez, Elaine R. Izcoa

**Affiliations:** 1 University of Puerto Rico-Medical Sciences Campus; 2 Tittle V Cooperative MSC-UCC

## Abstract

OBJECTIVES/SPECIFIC AIMS: Responding to the need and interest of students and faculty of the UHSP in learning about CTR, the Title V Cooperative Project between UPR-MSC and UCC, developed and offered a training cycle (TC) in CTR. METHODS/STUDY POPULATION: Undergraduate students (US), undergraduate faculty (UF), and graduate students (GS) were invited to register in: Research Education Towards Opportunities (RETO) and Mentorship Offering Training Opportunities for Research (MOTOR), which consisted of 20 hours of training in CTR, with interdisciplinary sessions in: Introduction and preparation of a presentation in CTR; Identify, interview and share a presentation of a CT researcher; participation in conferences and a summer camp in CTR. At the end of the TC, surveys—satisfaction and needs assessment—for training in CTR were administered. RESULTS/ANTICIPATED RESULTS: Thirty-three (33) registered in the TC, distributed: 13 (39.39%) US in RETO, 12 (36.36%) GS and 8 (24.24%) UF in MOTOR. Of these, 25 (75.75%) answered and submitted the on-line surveys and received a completion certificate. All (100%) were satisfied with the TC, and for 96% of the respondents, their expectations were fulfilled, and will continue in the TC. They selected critical review, scientific communication, and cultural diversity as thematic areas of interest. In addition, 60% of them selected neuroscience, cancer and medical imaging as main research areas of interest. DISCUSSION/SIGNIFICANCE OF IMPACT: The TC demonstrated to be an effective strategy to provide new knowledge, experiences, and interest in CTR. It also established a pathway for future engagement in CTR.

